# Corrigendum

**DOI:** 10.1016/s1674-8301(11)60012-2

**Published:** 2011-03

## Dear Editor:

On behalf of all the authors, I would like to correct some errors on page 412 (lines 7 to 13) of our article published on Journal of Biomedical Research (K Jin, SX Wang, ZH Huang, S Lu. Clostridium difficile infections in China. Journal of Biomedical Research, 2010; 24(6): 411-416): “In 1996, the rate of C. *difficile* infections in the United States hospitals was 31 cases/10 million patients; by 2003, this rate soared to 61 cases/10 million patients^[4]^. The study by the Association for Professionals in Infection Control and Epidemiology showed that the detection rate of C. *difficile* was 13.1% and the incidence was 12.4% in hospitalized patients, a significant increase over the previous data^[5]^” should be corrected as follows with the corrected information underlined “In 1996, the rate of C. *difficile* infections in the United States hospitals was 31 cases/100,000 patients; by 2003, this rate soared to 61 cases/100,000 patients^[4]^. The study by the Association for Professionals in Infection Control and Epidemiology showed that the detection rate of C. *difficile* was 13.1‰ and the incidence was 12.4‰ in hospitalized patients, a significant increase over the previous data^[5]^.”

We apologize for our mistakes and any inconvenience to readers.

Regards,

Ke Jin

On behalf of all authors

Department of Infectious Diseases/Jiangsu Province Key Laboratory in Infectious Diseases/China-US Vaccine Research Center, the First Affiliated Hospital of Nanjing Medical University, Nanjing, Jiangsu 210029, China

